# Metabolomic Method: UPLC-q-ToF Polar and Non-Polar Metabolites in the Healthy Rat Cerebellum Using an In-Vial Dual Extraction

**DOI:** 10.1371/journal.pone.0122883

**Published:** 2015-04-08

**Authors:** Amera A. Ebshiana, Stuart G. Snowden, Madhav Thambisetty, Richard Parsons, Abdul Hye, Cristina Legido-Quigley

**Affiliations:** 1 Institute of Pharmaceutical Sciences, King’s College London, Franklin-Wilkins Building, 150 Stamford Street, London, SE1 9NH, United Kingdom; 2 Institute of Psychiatry, Department of Old Age Psychiatry, King’s College London, De Crespigny Park, London, SE5 8AF, United Kingdom; 3 Clinical and Translational Neuroscience Unit, Laboratory of Behavioural Neuroscience, National Institute on Aging, Baltimore, Maryland, United States of America; Cleveland Clinic, UNITED STATES

## Abstract

Unbiased metabolomic analysis of biological samples is a powerful and increasingly commonly utilised tool, especially for the analysis of bio-fluids to identify candidate biomarkers. To date however only a small number of metabolomic studies have been applied to studying the metabolite composition of tissue samples, this is due, in part to a number of technical challenges including scarcity of material and difficulty in extracting metabolites. The aim of this study was to develop a method for maximising the biological information obtained from small tissue samples by optimising sample preparation, LC-MS analysis and metabolite identification. Here we describe an in-vial dual extraction (IVDE) method, with reversed phase and hydrophilic liquid interaction chromatography (HILIC) which reproducibly measured over 4,000 metabolite features from as little as 3mg of brain tissue. The aqueous phase was analysed in positive and negative modes following HILIC separation in which 2,838 metabolite features were consistently measured including amino acids, sugars and purine bases. The non-aqueous phase was also analysed in positive and negative modes following reversed phase separation gradients respectively from which 1,183 metabolite features were consistently measured representing metabolites such as phosphatidylcholines, sphingolipids and triacylglycerides. The described metabolomics method includes a database for 200 metabolites, retention time, mass and relative intensity, and presents the basal metabolite composition for brain tissue in the healthy rat cerebellum.

## Introduction

The brain is the centre of the nervous system in all vertebrates, and is responsible for controlling all bodily functions ranging from walking and talking, to heart rate and endocrine function. In addition to this diseases of the brain and central nervous system represent a major cause of global morbidity and mortality, with over 600 recognised neurological diseases [1] including developmental disorders such as Down syndrome and autism spectrum disorders [2–3], seizure disorders like epilepsy [4] and neurodegenerative disorders including Alzheimer’s and Parkinsons diseases [5–8]. Despite the importance of the brain and the pathological burden associated with it, we are still relatively ignorant of its mechanisms and it is hoped that developing a better understanding of cerebral metabolism will help to begin unlocking the secrets of the brain. Arguably, the biggest challenges of working with both human and animal brain tissue are twofold, firstly the small amounts/preciousness due to inaccessibility of sample material and secondly reproducible extraction of metabolites from the sample tissues. These obstacles make the development of analytical approaches that maximise the metabolites that can be reproducibly measured from small tissue samples an important challenge.

Metabolomics is the unbiased analysis of the composition of small molecule metabolites in a given biological tissue or fluid, under a specific set of environmental conditions [9–10]. Due to the wide range of concentrations at which these metabolites are present and their diverse physiochemical properties it is challenging to obtain comprehensive analysis of all metabolite classes using a single method [11–14]. Therefore many metabolomic approaches that aim to maximise metabolite coverage utilise a combination of analytical platforms including liquid chromatography—mass spectrometry (LC-MS), nuclear magnetic resonance (NMR) and gas chromatography—mass spectrometry (GC-MS) [11, 15–16]. These multi-platform approaches will measure metabolites with a wide range of concentrations and physiochemical properties, however the downside to increasing metabolite coverage will be a significant increase in the amount of tissue required.

LC-MS is one of the most widely used analytical techniques for metabolite fingerprinting and has been used to analyse a range of metabolite classes in a variety of biological matrices [17–20]. One of the major advantages of this approach is that it separates complex sample mixtures into its constituent components prior to mass spectral analysis. Separation enables the discrimination of some isobaric compounds which mass spectrometry alone cannot do, it also helps to reduce matrix effects in the ionisation chamber such as ionisation suppression in which different components of the matrix compete to be ionised resulting in a suppressed metabolite signal and incorrect metabolite quantitation [21–24]. However, one important limitation is that physiochemical properties of metabolites are diverse, and a single chromatographic technique cannot separate thousands of metabolites. For example reversed phase chromatography will separate non-polar metabolites such as lipids, but not separate polar compounds like amino acids [13]. This means that all of the polar metabolites will co-elute at the start of the chromatogram, with many not being measured correctly due to ion suppression. Therefore, as a result multiple chromatographic separation techniques are required to achieve a broad coverage of the metabolome. Sample preparation for LC-MS metabolite fingerprinting usually involves a solvent based (usually methanol, ethanol or acetonitrile) protein precipitation [25] to reduce surface absorption and protein-metabolite interactions. Different chromatographic conditions require distinct sample preparations increasing analysis time, analytical variability and the amount of sample material required. A main obstacle in metabolomics is metabolite identification, metabolite features measured need to be translated to chemical identities or metabolites that can give biological information.

Metabolite annotation has repeatedly been identified as a significant bottleneck in mass spectrometry untargeted workflows [26–27]. There are several challenges that make metabolite annotation difficult, the first of which is that there is up to an estimated 200,000 distinct metabolites [10] less than 50% of which have been structurally identified. Many metabolites, especially esoteric compounds, have unknown structure, so complete identification can only be done by compound synthesis, hence sharing of in-house databases is unusual. Secondly whilst fragmentation patterns are used for identification, this is an expert field and good quality fragmentation is not always possible.

To date there has been a number of metabolomic studies that have looked at the metabolite composition of brain tissue. Salek *et al*. [28] used 1H-NMR to measure the metabolite composition in the hippocampus, cortex, frontal cortex, midbrain and cerebellum of CRND8 mice identifying 23 metabolites from tissue samples ranging in mass from 10–50mg. In humans, brain tissue is in short supply and to date only small numbers (n = 10–15) with reversed phase fingerprinting have been profiled. However two groups were able to make important contributions, Graham *et al*. [29] used ≈5g of human post mortem brain and UPLC-ToF to develop a method that detected 1,264 metabolic features, with 10 features shown to be correlated to AD. Koichi *et al*. [30] also used UPLC-ToF metabolomics of human brain and found spermine and spermidine to be increased in AD pathology.

Therefore, this study aimed to obtain both polar and non-polar metabolites from a single small sample of brain tissue. For this HILIC together with reversed phase (RP) methods were investigated. Another aim was to provide the means for metabolite identification with the method, the data generated is the basal metabolome in rat cerebellum that can be applied in clinical investigations.

## Materials and Methods

### Chemicals and Reagents

All solvents, water, methanol, acetonitrile, ammonium formate, formic acid and methyl tertiary butyl ether (MTBE), were LC-MS grade purchased from Sigma-Aldrich. Four internal standards, heptadecanoic acid (≥ 98% purity), tripentadecanoin (≥ 98% purity) for the reversed phase, and L-serine^13^C_3_^15^N (95%) and L-valine^13^C_5_^15^N (95%) for HILIC were purchased from Sigma-Aldrich. In-vial dual extractions were performed in amber glass HPLC vials with fixed 0.4 mL inserts (Chromacol: Welwyn Garden City, UK).

### Samples

Experimental tissue material was obtained from the cerebellum of adult male (Sprague-Dawley) rats obtained from Harlan Laboratories UK. The animals were euthanized in the Biomedical services unit, King’s College London by inducing carbon dioxide (CO_2_) anoxia followed by cervical dislocation as per Schedule 1 of the Animal (scientific procedures) Act of 1986. All animal procedures were approved by local animal welfare and the Ethics Review Body (King’s College London). The cerebellum was isolated according to the Springer protocol for the dissection of rodent brain regions [31], samples were weighed and subsequently stored at -80°C. The cerebellum was sectioned on sterile glass slides (Thermo Scientific, Menzel-Glazer slides) using a sterile scalpel, both scalpel and slide were cooled in liquid nitrogen to reduce sample thawing during sectioning. Sectioned tissue samples were transferred to Eppendorf tubes containing a clean, pre-cooled, 5mm stainless steel ball bearing.

### Experimental Design

**Fig 1:**
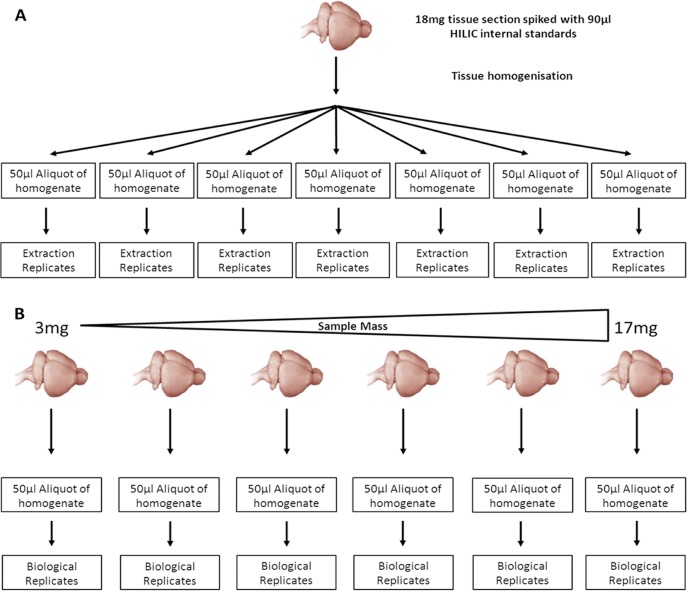
Graphical representation of the experimental designs used. A) experiment 1, a single 18mg brain section was homogenised then 7 parallel extractions were performed on 50μl aliquots of homogenate. B) Experiment 2, brain sections ranging from 3–17mg were homogenised and extracted parallel.

**Fig 2:**
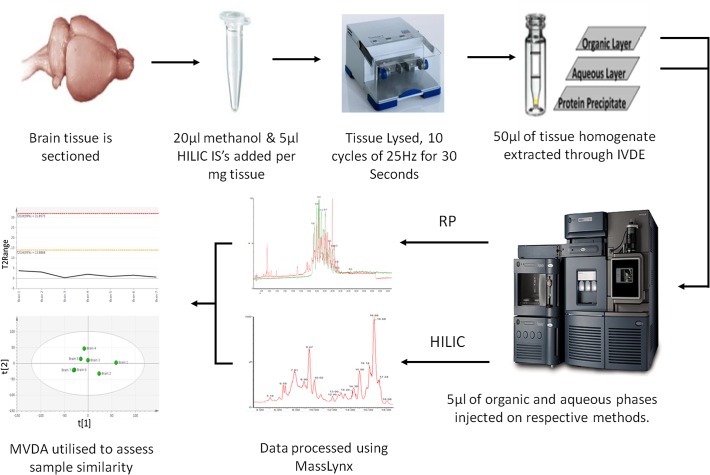
Applied analytical pipeline. Shows the seven steps from tissue sectioning to IVDE and onto data processing and multivariate analysis of variation.

In this study two primary experiments were performed to assess the precision and sensitivity of the IVDE, instrument methods and tissue homogenisation as well as to determine the effect of sample mass on metabolite recovery. The first experiment was designed to assess the combined variability of the IVDE and instrument methods. This was done by homogenising a single piece (18mg) of rat cerebellum, removing sample mass and tissue homogenisation as sources of variability. The homogenate was split into 7 aliquots of 50μl which underwent parallel extractions prior to injection on both HILIC and reversed phase methods (Fig 1A). The second experiment was designed to assess the effect of the mass of tissue extracted and tissue homegenistation on method sensitivity and precision. Four Sprague-Drawly rat brain were obtained and this material was used to perform Experiment 2. To do this 15 tissue samples ranging from 3–17mg were homogenised and extracted in parallel prior to analysis (Fig 1B). Sensitivity was assessed in terms of the number of metabolite features that are routinely detected, whilst precision will be assessed in terms of the variability (coefficient of variation) of the abundance of internal standard and metabolite peaks as well as the degree of compositional similarity between samples as determined principal component analysis (PCA). A graphical description of the analytical workflow used in this study is shown in Fig 2.

### Tissue homogenisation

Prior to homogenisation 20μl of methanol and 5μl of HILIC internal standard cocktail (2.5mM L-serine^13^C_3_^15^N and L-valine^13^C_5_^15^N in methanol:water (4:1)) was added per milligram of sample material. The tissue was then homogenised using a Tissuelyzer(Qiagen) in 10 cycles of 30 seconds at 25 Hz, subsequently a 50ul aliquot of homogenate was transferred to a Chromacol HPLC vial (400μl fixed insert).

### In-vial dual extraction of brain tissue

Subsequently 10μl of water was added to the homogenate, vials were then vortexed for 5 minutes, after which 250μl of MTBE containing Tripentadecanoin (10 μg/ml) and Heptadecanoic acid (10 μg/ml) was added after which samples were again vortexed at room temperature for 60 minutes. Following the addition of a further 40μl of water containing 0.15mM ammonium formate to enhance phase separation, samples were then centrifuged at 2500×g for 30 minutes at 4°C. This resulted in a clear separation of MTBE (upper) and aqueous (lower) phases, with protein precipitate aggregated at the bottom of the vial. Quality control samples were created by pooling excess tissue homogenate from biological samples (after a 50μl aliquot had been taken), this excess homogenate was then split into 50μl aliquots for in-vial extraction.

### LC-MS analysis of IVDE non-aqueous phase

LC-MS analysis was performed on a Waters Acquity ultra performance liquid chromatogram (UPLC) system coupled to a Waters Premier quadrupole time-of-flight (Q-Tof) mass spectrometer (Waters, Milford, MA, USA). The needle height in the auto-sampler was set to 13mm, with 5μl of sample extract injected onto an Agilent Poroshell 120 EC-C8 column (150mm × 2.1mm, 2.7 μm). Separation was performed at 55°C with a flow-rate of 0.5 ml/min using 10mM ammonium format in water (mobile phase A) and 10mM ammonium format in methanol (mobile phase B). For analysis in the positive mode, the gradient started at 80% mobile phase B increasing linearly to 96% B in 23 minutes and was held until 45 minutes then the gradient was increased to 100% by 46 minutes until 49 minutes. Initial conditions were restored in 2 minutes ahead of 7 minutes of column re-equilibration. For analysis in the negative ionisation mode the gradient started at 75% B increasing linearly to 96% B at 23 minutes, then increasing further to 100% B by 35 minutes, initial conditions were restored to allow 7 minutes of column re-equilibration. In the positive mode, a capillary voltage of 3.2 kV and a cone voltage of 45V was applied. Data was collected between 50 and 1000*m/z*, the desolvation gas flow was 400 L/hour and the source temperature was 120°C. In the negative mode, a capillary voltage of 2.6 kV and a cone voltage of 45 V were used. Desolvation gas flow and source temperature were fixed at 800 L/h and 350°C, respectively. All analyses were acquired using the lock spray to ensure accuracy and reproducibility; A reference solution (leucine-enkephalin) was used as lock mass (*m/z* 556.2771 and 278.1141) at a concentration of 200 ng/mL to update accurate mass data values and a flow rate of 10 μL/min. Data were collected in the centroid mode over the mass range *m/z* 50–1000 with an acquisition time of 0.1 seconds a scan.

### LC-MS analysis of IVDE aqueous phase

The auto sampler needle height was set at 2mm, with analysis of 5μl of aqueous phase extract being analysed on a Merck Sequant Zic-HILIC column (150 × 4.6mm, 5μm particle size) coupled to a Merck Sequant guard column (20 × 2.1mm). A 40 minute room temperature gradient (0.3ml/min) was applied using 0.1% formic acid in water (mobile phase A) and 0.1% formic acid in acetonitrile (mobile phase B). The gradient started at 80% mobile phase B, followed by a linear reduction to 20% mobile phase B after 30 minutes, initial conditions were restored to allow 10 minutes of column re-equilibration. Mass spectral data was acquired between 75–1000 Daltons in both positive and negative ionisation modes. The applied mass spectrometry conditions were the same as for the reversed phase method.

### Data processing and metabolite identification

The generated data was processed using MarkerLynx (Masslynx 4.1 Waters, USA) which provides automated peak detection based on peak alignment and normalization to total peak area. The reversed phase data were processed with a mass tolerance of 0.01 daltons (Da), a mass window of 0.05Da, and a retention time window of 12 seconds and a peak width of 10 seconds. The HILIC data was processed with a mass tolerance of 0.01 daltons (Da), a mass window of 0.05 Da, retention time window 18 seconds, and peak width of 20 seconds. Processed data was evaluated using principal component analysis (PCA) performed in SIMCA 13.0.3 (Umetrics, Umeå, Sweden). The data in all of the generated PCA models was logarithmically transformed (base 10) and scaled to unit variance (UV). The performance of the PCA models generated was assessed based on the cumulative correlation coefficients (R^2^X[cum]), and predictive performance based on seven-fold cross validation (Q^2^[cum]). Hotelling’s T^2^ plots were used to assess the departure of samples from the origin in the model plane, which will show the distance of a sample to a calculated average observation (i.e. an average metabolite composition). The DModX plots corresponds to the residual standard deviation of an observation in the x-variables, it was used to assess the distance of an observation to the fitted model.

Metabolite annotation was performed by searching the *m/z* of measured metabolite features in a range of publicly accessible metabolite databases including the human metabolome database (HMDB), METLIN and LipidMaps. Once potential metabolites had been identified it was confirmed by matching the fragmentation pattern of the peak being annotated to the fragmentation pattern shown for given metabolites in the literature and standard compounds. In addition some peaks in the reversed phase method were annotated by comparing the *m/z* and retention time of metabolite features to metabolite features previously annotated in Whiley *et al*. [32].

## Results/Discussion

### Assessing the effect of IVDE and LC-MS on method performance and precision (Experiment 1)

**Fig 3:**
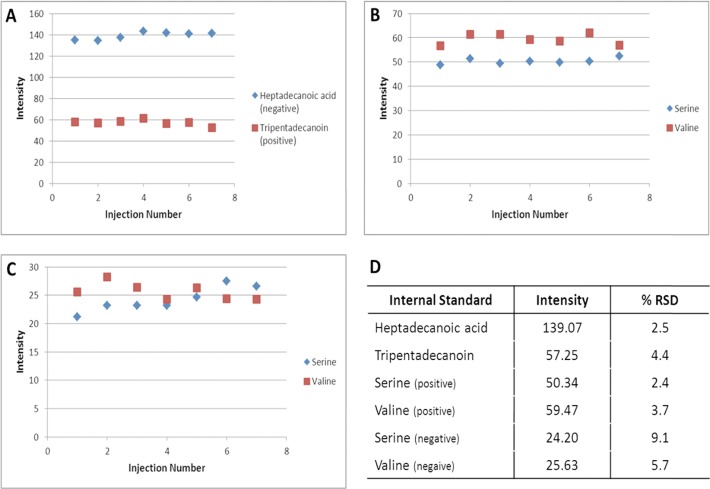
Recoveries of HILIC and reversed phase internal standards in experiment 1. A) plot of intensity of reversed phase internal standards Heptadecanoic acid (negative) and Tripentadecanoin (positive), B) plot of intensity of HILIC internal standards in positive ionisation mode, C) plot of intensity of HILIC internal standards in negative ionisation mode, D) average intensity and coefficient of variance of all internal standards.

The first step in assessing the precision of the in-vial dual extraction (IVDE) and both the reversed phase and HILIC methods was to determine the recovery for four internal standards (Fig 3). In the HILIC method both internal standards were measured in both the positive and negative ionisation modes. In the positive data the recovery of internal standards are highly consistent with coefficient of variation (CV) of 2.4% and 3.7% (Fig 3B) for the serine and valine standards respectively. In the negative mode, recovery is more variable than the positive mode with CV’s of 9.1% and 5.7% (Fig 3C) for serine and valine respectively. In the reversed phase method heptadecanoic acid was measured in the negative mode and tripentsdecanoin was measured in the positive. The recovery of both standards was consistent with CV’s of 2.5% and 4.4% for heptadecanoic acid and tripentadenanoin respectively. The standard recoveries suggests that the IVDE and both HILIC and reversed phase methods have good precision with all internal standard measurements having CV’s less than 15% [33], with mass spectrometry in the negative mode adding more variability than the positive mode.

The next step in determining the methods performance was to identify the number of metabolite features measured following HILIC and reversed phase separation and to assess the precision of these peaks. This was done by initially identifying the features present in all samples, then identifying those features measured in at least of 85% of samples, with a minimum cut off of peaks present in at least 70% of samples analysed (Tables 1 and 2). In total 5,841 metabolite features were measured in 100% of samples for both the HILIC (3713 metabolite features) and reversed phase (2128 metabolite features) methods. When a 70% sample presence cut off was applied, 12,274 metabolite features were identified with 6,570 and 5,704 metabolite features measured in the HILIC and reversed phase methods respectively. The measured metabolite features show good precision with 3,468 of the 5,841 (59.4%) of peaks seen in 100% of samples, and 6,362 of the 12,274 (51.8%) of the peaks measured in at least 70% of samples have CV’s of <15%. In general the features with CV’s of ≥15% are lower in abundance, with peaks at CV’s <15% with an average abundance 6.62 and peaks with CV’s ≥15% having an average of 1.93 potentially accounting for the lower precision. It is also interesting to note that the metabolite features that are measured in all samples have a higher average abundance (4.76) than those measured in 85% (2.04) and 70% (1.83). This is due to these groups possessing more peaks that are close to the limit of detection (LOD) with the peak falling below the LOD in some samples accounting for the missing values.

**Table 1:** Measured metabolite features in the HILIC method in experiment 1.

	HILIC Positive			HILIC Negative			HILIC Total		
%RSD	100%^a^	85%^a^	70%^a^	100%^a^	85%^a^	70%^a^	100%^a^	85%^a^	70%^a^
**< 5** ^**b**^	141	155	170	103	113	127	244	268	297
**5–10** ^**b**^	416	507	598	605	668	726	1021	1175	1324
**10–15** ^**b**^	305	436	582	460	618	726	765	1054	1308
**15–30** ^**b**^	417	635	889	803	1204	1539	1220	1839	2428
**> 30** ^**b**^	149	286	406	314	540	807	463	826	1213
**Total**	1428	2019	2646	2285	3143	3925	3713	5162	6570

Showing the number of metabolite peaks identified and their relative variability in 100%, 85% and 70% of 7 sample replicates.

^a^ percentage of samples a peak is detected in

^b^ coefficient of variance of peak intensity between samples.

**Table 2:** Measured metabolite features in the reversed phase method in experiment 1.

	RP Positive			RP Negative			RP Total		
%RSD	100%^a^	85%^a^	70%^a^	100%^a^	85%^a^	70%^a^	100%^a^	85%^a^	70%^a^
**< 5** ^**b**^	124	261	278	202	253	455	326	514	733
**5–10** ^**b**^	193	418	450	504	628	1112	697	1046	1562
**10–15** ^**b**^	69	115	197	346	418	941	415	533	1138
**15–30** ^**b**^	112	246	329	402	484	1009	514	730	1338
**> 30** ^**b**^	50	134	226	126	340	707	176	474	933
**Total**	548	1174	1480	1580	2123	4224	2128	3297	5704

Showing the number of metabolite peaks identified and their relative variability in 100%, 85% and 70% of 7 sample replicates.

^a^ percentage of samples a peak is detected in

^b^ coefficient of variance of peak intensity between samples.

**Fig 4:**
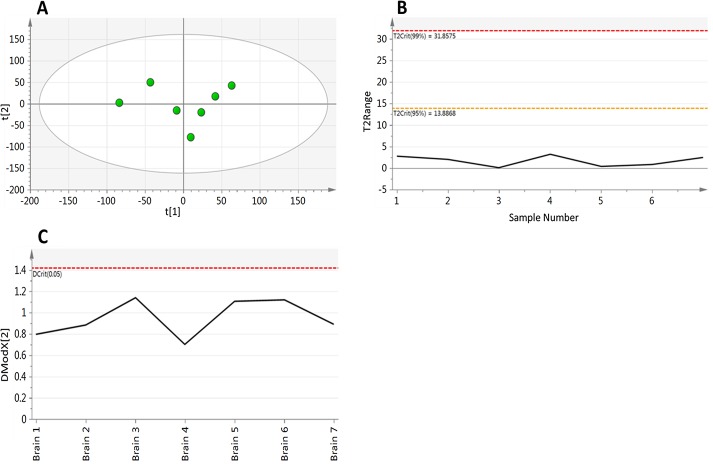
Principal component analysis (components = 2, R^2^X – 0.439, Q^2^–0.210) of metabolite features identified in at least 70% of samples in experiment 1. A) scores plot and B) Hotelling’s T^2^ and C) DModX plot, showing that sample mass has no effect on overall metabolite composition.

Having considered the behaviour of individual metabolite peaks, the final step in assessing the method performance is to look at the similarity of the overall composition of the analysed samples. Principal component analysis (PCA) was performed on all 12,274 metabolite features that were identified in at least 70% of samples (Fig 4). This PCA revealed little structure within the data with the first component accounting for only 25.3% of the total variability with a predictive performance of Q^2^ = -0.10, with the first two components accounting for just 43.9% of variability with a predictive performance of Q^2^ = -0.21. The distance of a samples metabolite composition to a calculated average composition was assessed using the Hotelling’s T^2^ range plot (Fig 4B). This plot shows that all of the samples are compositionally similar both to each other and the calculated average, with all samples having a T^2^ of < 5 with the 95% confidence interval set at 13.88. The distance of samples to the model was assessed using the DModX plot (Fig 4C), which shows that the samples have a low residual of difference to the fitted model with all of the observations falling below the Dcritical(0.05) threshold. This combined with the Hotelling’s T^2^ show that all of the samples are compositionally similar and that there are no outliers to the model.

### Assessing the effect of tissue homogenisation and sample mass on method performance and precision (Experiment 2)

**Fig 5:**
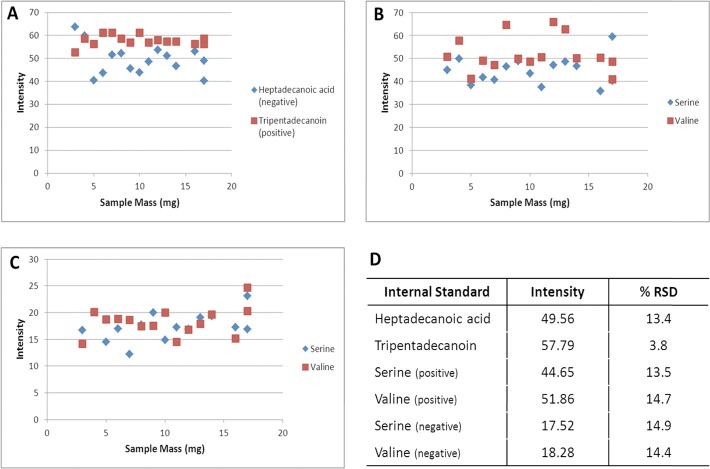
Recoveries of HILIC and reversed phase internal standards in experiment 2. A) plot of intensity of reversed phase internal standards Heptsdecanoic acid (negative) and Tripentadecanoin (positive), B) plot of intensity of HILIC internal standards in positive ionisation mode, C) plot of intensity of HILIC internal standards in negative ionisation mode, D) average intensity and coefficient of variance of all internal standards.

As with assessing the performance of the IVDE and instrument methods, the first step in assessing the effect of tissue homogenisation and sample mass is to look at the recovery of the internal standards. As in experiment 1 both HILIC internal standards are seen in positive and negative ionisation modes (Fig 5B and 5C). In the positive mode the CV’s of the internal standard recoveries were 13.5% and 14.7% for serine and valine respectively. In the negative mode CV’s of the internal standard recoveries were 14.9% and 14.4% for serine and valine respectively. In the reversed phase data heptadecanoic acid is measured in the negative mode with a CV of 13.4%, and tripentadecanoin was measured in the positive mode with a CV of 3.8%. The recovery of the HILIC internal standards is more variable in these samples than in experiment 1, suggesting that the tissue homogenisation step is contributing significantly to analytical variability. This is further supported by no increase in the variability of tripentadecanoin which is spiked into the sample after tissue homogenisation. The recovery of the HILIC internal standards in the quality control samples, which are pooled after tissue homogenisation, were more consistent than in the analytical samples, and comparable with experiment 1 with CV’s of 3.8% and 4.8% in positive and 5.3% and 7.1% in negative for serine and valine respectively, further supporting the hypothesis that tissue homogenisation is contributing significantly to the observed variability. With the increased CV’s showing that tissue homogenisation is contributing to an increase in data variability, it is important to assess the effect of the extracted tissue volume on the recovery of the internal standards. Spearman’s correlation was used to assess the relationship between standard recovery and sample mass, this analysis revealed no significant correlations showing that internal standard recovery is independent of the sample mass extracted.

The next step in assessing the method performance is to determine the number of metabolite features measured and the precision of these peaks. As in experiment 1 this was initially done by identifying peaks that were measured in all samples, working down to a cut off of peaks present in at least 73% of samples. In total 4,021 peaks were measured in 100% of samples, with 2,838 and 1,183 measured in HILIC (Table 3) and reversed phase (Table 4) methods respectively, 10,934 peaks measured in 73% of samples with 6,737 and 4,197 measured in HILIC and reversed phase data respectively. The precision of the measured peaks is lower than was seen in experiment 1 with 1,726 of 4,021 (43.7%) of the peaks seen in 100% of samples and 3,151 of 10,934 (28.8%) of peaks seen in 70% of samples having CV’s of <15%. The finding of higher sample to sample variability of the measured metabolite features lends further support to the hypothesis of tissue homogenisation as a source of variability within the method. A transformation of the HILIC data to correct for the variability introduced during tissue homogenisation was performed by normalising peak intensity to an average of the abundance of the two internal standards, however this correction did not improve precision of the measured metabolite peaks (S1 Table).

**Table 3:** Measured metabolite features in the HILIC method in experiment 2.

	HILIC Positive					HILIC Negative					HILIC Total				
**%RSD**	**100%** ^a^	**93%** ^a^	**87%** ^a^	**80%** ^a^	**73%** ^a^	**100%** ^a^	**93%** ^a^	**87%** ^a^	**80%** ^a^	**73%** ^a^	**100%** ^a^	**93%** ^a^	**87%** ^a^	**80%** ^a^	**73%** ^a^
**< 5** ^**b**^	38	49	56	68	81	33	39	44	55	61	71	88	100	123	142
**5–10** ^**b**^	322	408	431	467	509	217	302	283	344	372	539	710	714	811	881
**10–15** ^**b**^	426	566	601	644	685	222	353	386	495	566	648	919	987	1139	1251
**15–30** ^**b**^	406	583	742	793	884	501	751	1115	1142	1348	907	1334	1857	1935	2232
**> 30** ^**b**^	454	610	827	1078	1254	219	362	514	735	977	673	972	1341	1813	2231
**Total**	1646	2216	2657	3050	3413	1192	1807	2342	2771	3324	2838	4023	4999	5821	6737

Showing the number of metabolite peaks identified and their relative variability in 100%, 93%, 87%, 80% and 73% of 15 sample replicates.

^a^ percentage of samples a peak is detected in

^b^ coefficient of variance of peak intensity between samples.

**Table 4:** Measured metabolite features in the reversed phase method in experiment 2.

	RP Positive					RP Negative					RP Total				
%RSD	100%^a^	93%^a^	87%^a^	80%^a^	73%^a^	100%^a^	93%^a^	87%^a^	80%^a^	73%^a^	100%^a^	93%^a^	87%^a^	80%^a^	73%^a^
**< 5** ^**b**^	168	184	195	229	238	9	9	14	14	14	177	193	209	243	252
**5–10** ^**b**^	203	213	220	231	235	7	11	30	38	38	210	224	250	269	273
**10–15** ^**b**^	65	72	93	140	156	46	81	127	191	196	111	153	220	331	352
**15–30** ^**b**^	103	119	147	166	182	267	271	754	1314	1363	370	390	901	1480	1545
**> 30** ^**b**^	49	62	89	135	184	266	388	901	1524	1591	315	450	990	1659	1775
**Total**	588	650	744	901	995	595	760	1826	3081	3202	1183	1410	2570	3982	4197

Showing the number of metabolite peaks identified and their relative variability in 100%, 93%, 87%, 80% and 73% of 15 sample replicates.

^a^ percentage of samples a peak is detected in

^b^ coefficient of variance of peak intensity between samples.

**Fig 6:**
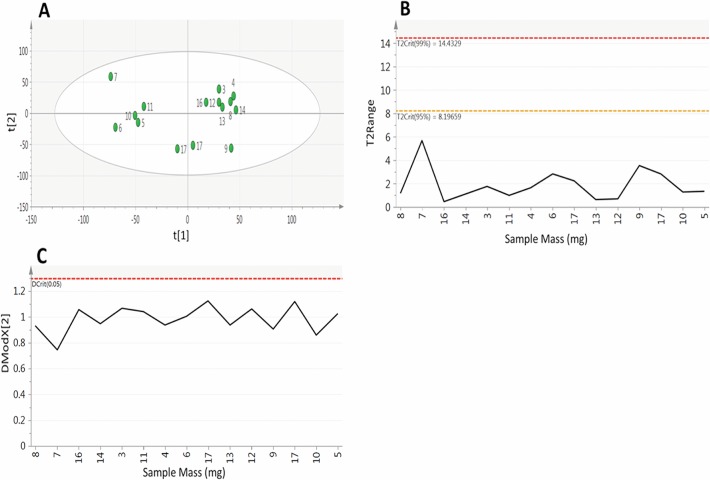
Principal component analysis of samples (components = 2, R^2^X 0.354, Q^2^ 0.049) performed on metabolite features identified in at least 73% of samples in experiment 2. A) scores plot where point labels represent sample mass B) Hotelling’s T^2^ and C) DModX plot of analytical samples, showing that sample mass has no effect on overall metabolite composition.

Having considered metabolite features individually it is important to consider the composition of samples as a whole. As in experiment 1 PCA was applied to all metabolite features that were measured in at least 73% of samples (Fig 6). The analysis revealed little structure within the data with the first component accounting for only 22.3% of total variability with a poor predictive performance of Q^2^ = 0.07, with the second component only explaining a further 13.1% of variability (Q^2^ = 0.05) (Fig 6A). The Hotelling’s T^2^ plot (Fig 6B) shows that all samples fall within the 95% confidence interval (T^2^ = 8.19), with all bar one sample having a T^2^ < 4 demonstrating that the samples are compositionally similar both to each other and to the calculated average. The DModX plot (Fig 6C) shows that all samples have a low residual of difference to the fitted model with all of the observations falling below the Dcritical(0.05) threshold. This combined with the Hotelling’s T^2^ plot show that all samples are compositionally similar and that there are no outliers to the model.

**Fig 7:**
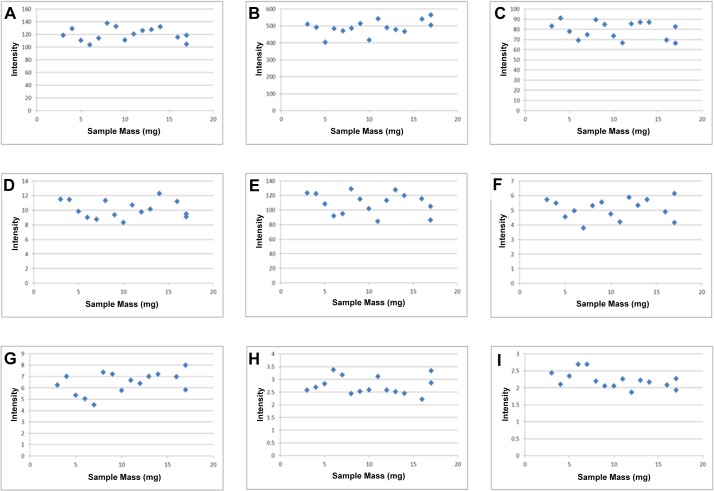
Plots of sample mass in milligrams against intensity for 9 annotated metabolites. A) taurine B) hypoxanthine C) glutamate D) pantothenate E) aspartate F) glcosylceramide (36:1) G) phosphatidylethanolamine (38:4) H) ceramide (38:1) I) triglyceride (48:3).

Whilst all samples are compositionally similar it is important to determine the effect of the extracted tissue mass on metabolite composition. Looking at the PCA scores plot (Fig 6A) it can be seen that there is no bias in the distribution of samples based on the tissue mass, with low and high mass samples clustering together within the plot showing that they possess high levels of compositional similarity. As well as looking at the effect of sample mass on the compositional similarity it is important to assess its effect on the abundance of individual metabolites. Fig 7 shows the abundance of 9 annotated metabolites from both HILIC and reversed phase methods plotted against the tissue mass, these plots show no relationship between metabolite abundance and sample mass, with the strongest correlation being for glutamate (r = -0.24). This data shows that using between 3–17mg of sample material has no effect on the overall sample composition or the abundance of individual metabolites, showing this method can provide broad metabolite coverage when sample material is limited.

### Annotated metabolites

Having optimised the sensitivity and reproducibility of the metabolite features measured by the analytical method, the final step is to demonstrate its biological relevance by linking the data directly to metabolism by annotating metabolites from a variety of chemical classes and across a range of concentrations. To do these 200 metabolites, 100 from both the HILIC and reversed phase methods were annotated (Tables 5 and 6). The annotated metabolites come from a wide range of metabolite classes including amino acids, purines, phospholipids and glycerides, across 3.5 orders of magnitude ranging in abundance from 0.1 to 576.9. There is limited overlap between the two analytical methods with no identified metabolites in common, this limited overlap demonstrates the necessity of using complimentary separation techniques like HILIC and reversed phase chromatography to obtain a comprehensive view of all of chemical space. These annotations enable the method to be easily compared as basal metabolite abundance in the rat’s healthy cerebellum and provide valuable information allowing the method to be accurately replicated by other laboratories.

**Table 5:** Metabolites annotated from the HILIC method.

Name	Formula	Molecular Weight (Da)	Retention time (Mins)	Intensity	
				Positive	Negative
Acetylalanine	C5H9NO3	131.0582	15.56	6.2	-
Acetylaspartate	C6H9NO5	175.0480	7.66	6.4	56.2
Acetylaspartylglutamate	C11H16N2O8	304.0906	8.38	7.2	28.6
Acetylcarnitine	C9H17NO4	203.1157	14.08	27.0	0.4
Acetylneuraminate	C11H19NO9	309.1059	23.88	0.4	-
Acetylserine	C5H9NO4	147.0531	8.31	-	22.3
Adenosine	C10H13N5O4	267.0967	12.92	33.3	-
Adenosine monophosphate	C10H14N5O7P	347.0630	15.60	6.2	-
Adrenaline	C9H13NO3	183.0895	6.74	2.9	-
Alanine	C3H7NO2	89.0476	16.61	184.5	39.2
Aminobutyrate	C4H9NO2	103.0633	16.25	-	84.5
Arachidonoyl glycidol	C23H36O3	360.2664	5.01	7.8	-
Arginine	C6H14N4O2	174.1116	26.47	40.9	7.4
Ascorbate	C6H8O6	176.0320	10.72	4.2	69.1
Asparagine	C4H8N2O3	132.0534	17.01	-	1.4
Aspartate	C4H7NO4	133.0375	7.81	109.2	33.6
Butyrlcarnitine	C11H21NO4	231.1470	12.11	1.7	-
Carnitine	C7H15NO3	161.1051	17.06	135.5	-
Carnosine	C9H14N4O3	226.1065	28.8	2.1	-
Citrate	C6H8O7	192.0270	10.7	-	1.2
Citrulline	C6H13N3O3	175.0956	17.17	2.7	2.2
Coumarate	C9H8O3	164.0473	14.40	26.7	-
Creatine	C4H9N3O2	131.0694	16.62	576.9	20.5
Creatinine	C4H7N3O	113.0589	16.61	15.6	1.0
Cystathionine	C7H14N2O4S	222.0674	21.74	7.4	1.2
Cysteine	C3H7NO2S	121.0197	15.30	2.8	-
Cytidine	C9H13N3O5	243.0855	17.33	2.1	1.2
Deoxyfluorouridine	C9H11FN2O5	246.0651	12.20	-	6.2
Dimethylarginine	C8H18N4O2	202.1429	24.45	1.4	-
Dimethylglycine	C4H9NO2	103.0633	17.21	24.9	-
Fumarate	C4H4O4	116.0109	7.78	-	0.8
Gluconate	C6H12O7	196.0583	15.64	-	43.1
Glutamate	C5H9NO4	147.0531	16.17	79.1	61.6
Glutamine	C5H10N2O3	146.0691	16.78	27.9	95.2
Glutamyl-glutamate	C10H14N2O7	274.0801	15.61	8.2	-
Glutamyl-leucine	C11H19N2O5	259.1293	15.48	2.8	-
Glutathione	C10H17N3O6S	307.0838	15.06	22.8	-
Glycerophosphatidylcholine	C8H20NO6P	257.1028	18.06	42.4	-
Glycine	C2H5NO2	75.0320	17.13	2.2	1.3
Glycolate	C2H4O3	76.0160	6.51	-	4.5
Guanidinobutanoate	C5H11N3O2	145.0851	15.73	8.4	-
Guanine	C5H5N5O	151.0494	11.90	13.5	7.9
Guanosine	C10H13N5O5	283.0916	11.87	2.3	18.4
Hexose-deoxy sugar	C6H12O5	164.0679	14.39	-	5.2
Hexose-Phosphate	C6H13O9P	260.0297	14.94	-	17.4
Histidine	C6H9N3O2	155.0694	25.13	14.6	6.1
Hydroxyphenylglycine	C8H9NO3	167.0582	16.61	-	0.7
Hydroxyproline	C5H9NO3	131.0582	16.17	-	1.6
Hypoxanthine	C5H4N4O	136.0385	9.45	489.9	108.7
Indoleacetate	C10H9NO2	175.0633	9.17	5.0	-
Inosine	C10H12N4O5	268.0807	10.03	45.7	388.8
Kynurenine	C10H12N2O3	208.0847	12.19	0.9	-
Lactate	C3H6O3	90.0316	7.57	-	6.1
leucine/Isoleucine	C6H13NO2	131.0946	12.69	20.3	1.6
Lysine	C6H14N2O2	146.1055	26.62	4.7	16.3
Lysophosphatidylserine	C24H48NO9P	326.3033	6.89	4.9	-
Malate	C4H6O5	134.0215	8.66	-	9.5
Malonate	C3H4O4	104.0109	7.27	0.4	-
Methionine	C5H11NO2S	149.0510	13.43	7.3	1.2
Methyladenosine	C11H15N5O4	281.1124	17.16	0.9	0.7
Methylaspartate	C5H9NO4	147.0531	8.39	48.4	20.3
Methylbutyroylcarnitine	C12H23NO4	245.1627	11.8	0.3	-
Methylfuranone	C6H8O2	98.0368	15.66	-	223.4
Methylhistidine	C7H11N3O2	169.0851	25.54	2.2	-
Methylsulfolene	C5H8O2S	132.0245	13.44	16.9	-
Methylthioadenosine	C11H15N5O3S	297.0895	10.61	1.3	-
Methylthiophene	C5H6S	98.0190	7.80	1.2	-
Myo-inositol	C6H12O6	180.0633	15.83	5.3	5.0
Nicotinamide	C6H6N2O	122.0480	9.57	104.8	-
Nicotinate	C6H5NO2	123.0320	9.14	-	0.6
Ornithine	C5H12N2O2	132.0898	26.39	0.5	14.4
Oxoproline	C5H7NO3	129.0425	16.77	217.42	30.2
Pantothenate	C9H17NO5	219.1106	6.74	10.1	2.2
Pentose sugar	C5H10O5	150.0528	13.55	-	10.7
Phenylalanine	C9H11NO2	165.0789	12.03	9.2	0.4
Phosphatidylcholine	C40H80NO8P	733.5621	9.08	63.6	-
Phosphenolpyruvate	C3H5O6P	167.9823	12.09	-	0.4
Phosphocreatine	C4H10N3O5P	211.0358	15.05	4.1	-
Pipecolate	C6H11NO2	129.0789	14.76	1.7	-
Proline	C5H9NO2	115.0633	14.99	30.2	0.3
Propionylcarnitine	C10H19NO4	217.1314	12.92	1.3	-
Putrescine	C4H12N2	88.1000	34.8	-	0.8
Riboflavin	C17H20N4O6	376.1382	7.9	5.5	-
Serine	C3H7NO3	105.0425	17.06	4.2	1.9
Spermidine	C7H19N3	145.1578	30.46	1.1	-
Taurine	C2H7NO3S	125.0146	15.00	119.9	286.3
Thiouracil	C4H4N2OS	128.0044	8.4	-	0.9
Threonine/Homoserine	C4H9NO3	119.0582	16.37	6.9	-
Thymidine	C10H14N2O5	242.0902	8.2	-	1.6
Thymine	C5H6N2O2	126.0429	9.30	0.6	25.6
Tocopherol	C29H50O2	430.3810	20.89	5.1	-
Tryptophan	C11H12N2O2	204.0898	12.74	4.2	1
Tyrosine	C9H11NO3	181.0738	14.39	8.8	14.9
Uracil	C4H4N2O2	112.0272	9.14	27.3	6.3
Urate	C5H4N4O3	168.0283	11.19	2.3	3.8
Uridine	C9H12N2O6	244.0695	9.18	1.7	12.1
Valine	C5H11NO2	117.0789	14.66	4.6	0.9
Xanthine	C5H4N4O2	152.0334	8.88	78.1	51.7
Xanthosine	C10H12N4O6	284.0756	11.85	-	2.8
Xanthurenate	C10H7NO4	205.0375	11.20	1.4	-

Annotations were made by matching fragmentation of analyte peaks to fragmentations in publicly accessible databases. Displaying the molecular formula, molecular weight in daltons, retention time in minutes and intensity in positive and negative ionisation modes in arbitrary units for all metabolites.—represents metabolites not detected in this ionisation mode.

**Table 6:** Metabolites annotated from the reversed phase method.

Name	Formula	Molecular Weight (Da)	Retention time (Mins)	Intensity	
				Positive	Negative
Docosahexaenoic acid	C22H32O2	328.2400	5.02	-	16.8
Hexadecynyl acetate	C18H32O2	280.2388	4.61	-	1.7
Hydroxycholestanol	C27H48O2	404.3636	15.06	-	3.7
DG(40:5)	C43H74O5	670.5536	20.79	1.8	-
DG(34:2)	C37H68O5	592.5066	27.07	0.7	-
DG(42:3)	C45H82O5	702.6162	17.58	3.9	-
DG(37:2)	C40H74O5	634.5536	27.14	1.2	-
SM(d34:1)	C39H80N2O6P	703.5753	13.20	0.7	-
SM(41:2)	C46H92N2O6P	799.6693	25.84	4.6	
SM(d41:1)	C46H94N2O6P	801.6849	18.55	1.9	-
SM(d42:1)	C47H96N2O6P	815.7006	24.13	3.9	-
SM(d43:2)	C48H96N2O6P	827.7006	20.20	18.9	-
Creatine 16:1 OH	C39H77N2O7P	716.5468	16.53	-	10.2
TG(52:7)	C55H92O6	848. 6893	28.10	6.2	-
TG(52:3)	C55H100O6	856.7519	19.44	1.4	-
TG(48:3)	C51H92O6	800.6823	17.70	2.3	-
TG(50:3)	C53H96O6	828.7112	19.97	1.1	-
TG(54:7)	C57H96O6	876.7199	30.36	3.1	-
TG(54:6)	C57H98O6	878.7420	30.43	1.1	-
TG(56:7)	C59H98O6	902.7421	32.71	1.2	-
PI(34:1)	C43H81O13P	836.5414	12.32	73.1	-
PI(36:3)	C45H81O13P	860.5414	21.29	-	6.93
PI(32:0)	C41H79O13P	810.5258	20.97	-	19.00
PI(38:2)	C47H87O13P	891.1596	19.08	-	34.13
PI(40:5)	C49H85O13P	912.5727	16.31	3.2	
PI(38:5)	C_47_H_81_O_13_P	884.5414	23.33	7.2	-
PA(36:2)	C39H73O8P	700.9659	15.97	1.4	-
PA(34:1)	C37H71O8P	674.9286	20.68	32.8	-
PA(39:0)	C42H85O7P	732.6091	14.89	0.9	-
PA(34:2)	C37H69O8P	672.4730	20.33		
GluCer(36:1)	C42H81NO8	727.5962	24.19	5.2	-
GluCer(d40:1)	C46H89NO8	783.6588	23.02	16.7	-
GluCer(d40:2)	C46H87NO8	781.6351	23.60	1.1	4.3
GluCer(42:2)	C48H91NO8	809.6744	19.18	1.8	-
PC(31:2)	C39H74NO8P	715.5134	18.85	2.4	-
PC(36:2)	C44H84NO7P	770.7432	18.36	1.5	-
PC(33:2)	C41H78NO8P	743.5492	20.21	1.7	12.1
PC(32:3)	C42H74NO8P	751.5152	18.77	-	57.7
PC(33:1)	C41H80NO8P	745.5574	21.30	-	0.3
PC(37:6)	C45H78NO8P	791.5417	19.61	-	1.9
PC(32:2)	C40H76NO8P	729.5322	15.98	13.7	-
PC(38:9)	C46H74NO8P	799.5298	19.11	-	45.4
PC(35:0)	C43H86NO8P	775.6091	15.75	0.71	
PC(35:6)	C43H74NO8P	763.5146	18.70	2.3	22.4
PC(35:3)	C43H80NO8P	769.5688	20.70	41.5	-
PC(35:6)	C43H74NO8P	763.5146	17.63	2.1	-
PC(35:3)	C43H80NO8P	769.5688	25.57	1.5	-
PC(36:3)	C44H82NO8P	783.5663	15.49	15.7	-
PC(P-38:5)	C46H82NO7P	791.5828	14.78	0.5	-
PC(44:1)	C52H103NO8P	900.7242	24.05	19.9	-
PC(P-38:6)	C46H80NO7P	789.5609	18.20	1.0	-
PC(44:0)	C52H105NO8P	902.7421	28.94	18.6	-
PC(45:0)	C53H107NO8P	916.6589	27.84	11.5	-
PC (38:0)	C46H92NO8P	817.6561	26.70	3.2	-
PC (40:1)	C48H94NO8P	843.6717	20.24	6.6	6.4
PC (40:6)	C48H84NO8P	833.5935	18.78	1.5	68.1
PC (38:6)	C44H76NO8P	805.5622	15.60	16.8	-
PC (40:2)	C48H92NO8P	841.6560	21.07	-	3.6
PG(34:1)	C40H77O10P	748.5254	14.43	3.0	-
PG(38:3)	C44H81O10P	800.5567	17.50	14.8	-
PG(36:2)	C42H79O10P	774.5410	17.53	1.4	-
PG(42:0)	C48H97O9P	848.6842	19.75	2.7	-
PE(35:0)	C40H80NO8P	733.5621	13.31	1.3	
PE(40:6)	C45H78NO8P	791.5417	19.61	-	31.3
PE(33:1)	C38H74NO8P	703.5158	16.54	24.6	-
PE(35:2)	C40H80NO8P	729.5308	25.83	4.9	-
PE(38:3)	C43H80NO8P	769.5688	25.96	24.2	-
PE(33:2)	C38H72NO8P	701.4996	18.18		36.9
PE(34:2)	C39H74NO8P	715.5134	18.85	-	12.4
PE(36:4)	C41H76NO8P	739.5154	12.76	0.1	-
PE(36:2)	C41H78NO8P	743.5492	14.70	2.4	-
PE(36:5)	C41H72NO8P	737.4995	21.03	2.5	-
PE(36:1)	C41H76NO8P	745.5574	20.23	-	0.3
PE(38:6)	C43H74NO8P	763.5156	18.70	12.2	-
PE(38:4)	C43H78NO8P	768.0551	23.40	6.4	-
PE(46:1)	C51H100NO8P	885.7367	22.07	7.6	-
PE(O-36:0)	C41H86NO6P	719.6105	12.32	0.1	-
PE(40:2)	C45H86NO8P	799.6091	16.03	1.0	-
PE(44:8)	C49H82NO8P	844.5151	19.64	3.2	-
LPC(18:2)	C26H50NO7P	519.3324	12.10	2.8	
LPC(20:4)	C28H50NO7P	543.3353	11.80	3.1	-
LPC(24:1)	C32H64NO7P	605.4384	9.13	2.1	-
LPA(18:0)	C21H43O7P	438.2746	15.97	0.9	
LPE(22:0)	C27H56NO7P	537.7098	15.76	-	10.1
LPE(24:1)	C29H58NO7P	563.7471	16.41	-	7.2
PS(30:1)	C36H68NO10P	705.4580	18.33	0.3	-
PS(38:3)	C44H80NO10P	813.5519	21.55	-	1.9
PS(40:5)	C46H80NO10P	837.5519	12.44	0.7	-
PS(37:3)	C43H78NO10P	799.5298	10.64	-	1.5
PS(36:1)	C42H80NO10P	789.5609	13.75	0.8	-
PS(36:0)	C42H82NO10P	791.5800	14.77	0.4	-
PS(36:5)	C42H72NO10P	781.9955	12.70	1.1	-
PS(0–34:0)	C40H80NO9P	749.5511	14.55	0.3	-
PS(34:1)	C40H76O10P	761.5975	16.88	1.3	-
Cer(36:1)	C36H67NO3	565.5435	17.49	-	8.6
Cer(38:1)	C38H67NO3	593.5745	19.16	-	2.7
Cer(40:1)	C40H67NO3	621.6163	20.60	-	1.9
Cer(42:1)	C42H83NO3	649.6372	20.83	-	1.2
Cer(d44:2)	C44H85NO3	675.6477	24.30	5.6	-
Cer(d40:1)	C40H80NO6P	701.5575	15.13	3.8	-

Annotations were made by matching fragmentation of analyte peaks to fragmentations in publicly accessible databases. Displaying the molecular formula, molecular weight in daltons, retention time in minutes and intensity in positive and negative ionisation modes in arbitrary abundance units for all metabolites.—represents metabolites not detected in this ionisation mode. **Abbreviations:** Lysophosphatidylcholines (LPC), Lysophosphatidylethanolamines (LPE),Phosphatidic acids (PA), Phosphatidylcholines (PC), Ether-linked phosphatidylethanolamines (PE-O), Phosphatidylglycerols (PG), Phosphatidylinositols (PI), Phosphatidylserines (PS), Ether-linked phosphatidylserines(PS-O), Ether-linked phosphatidylserines (PS-O), Sphingomyelins (SM), Dihydroxy-glucosylceramide (GluCer-d), Dihydroxyceramide (Cer-d), Triacylglycerols (TG), Diacylglycerols (DG).

## Conclusions

The method described in this paper is shown to be capable of measuring over 4,000 metabolite features from as little as 3mg of tissue with a high degree of reproducibility of which we were able to annotate 200 metabolites from a variety of metabolite classes across a range of concentrations. It is hoped that the low required sample mass and improved sensitivity of this method will provide a valuable tool to analyse cerebral metabolism, hopefully providing new insights into the functioning of the brain as well as the mechanisms of pathology of neurological disorders.

## Supporting Information

S1 Table Measured metabolite features in the HILIC method in experiment 2. Showing the number of metabolite peaks identified and their relative variability in 100%, 93%, 87%, 80% and 73% of 15 sample replicates after transformation based on the recovery of both internal standards. ^a^ percentage of samples a peak is detected in, ^b^ coefficient of variance of peak intensity between samples.
(DOCX)
