# Supplementary material for: Metabolomic Method: UPLC-q-ToF Polar and Non-Polar Metabolites in the Healthy Rat Cerebellum Using an In-Vial Dual Extraction
Source: PLoS One. 2015 Apr 8;10(4):e0122883. doi: 10.1371/journal.pone.0122883 (PMC4390242; doi:10.1371/journal.pone.0122883)
Supplement: S1 Table — Showing the number of metabolite peaks identified and their relative variability in 100%, 93%, 87%, 80% and 73% of 15 sample replicates after transformation based on the recovery of both internal standards. a percentage of samples a peak is detected in, b coefficient of variance of peak intensity between samples. (DOCX) [file pone.0122883.s001.docx]

Supplemental Table 1. **Measured metabolite features in the HILIC method in experiment 2.** Showing the number of metabolite peaks identified and their relative variability in 100%, 93%, 87%, 80% and 73% of 15 sample replicates after transformation based on the recovery of both internal standards. ^a^ percentage of samples a peak is detected in, ^b^ coefficient of variance of peak intensity between samples.

|  | **HILIC Positive** | | | | | **HILIC Negative** | | | | | **HILIC Total** | | | | |
| --- | --- | --- | --- | --- | --- | --- | --- | --- | --- | --- | --- | --- | --- | --- | --- |
| **%RSD** | **100%**^a^ | **93%**^a^ | **87%**^a^ | **80%**^a^ | **73%**^a^ | **100%**^a^ | **93%**^a^ | **87%**^a^ | **80%**^a^ | **73%**^a^ | **100%**^a^ | **93%**^a^ | **87%**^a^ | **80%**^a^ | **73%**^a^ |
| **< 5^b^** | 41 | 47 | 58 | 71 | 88 | 29 | 45 | 45 | 51 | 57 | 70 | 92 | 103 | 122 | 145 |
| **5-10^b^** | 327 | 414 | 421 | 474 | 523 | 237 | 321 | 327 | 363 | 387 | 564 | 735 | 748 | 837 | 910 |
| **10-15^b^** | 421 | 555 | 614 | 681 | 660 | 208 | 339 | 373 | 475 | 541 | 629 | 894 | 987 | 1156 | 1201 |
| **15-30^b^** | 397 | 577 | 737 | 771 | 900 | 481 | 733 | 1135 | 1153 | 1377 | 878 | 1310 | 1872 | 1924 | 2277 |
| **> 30^b^** | 460 | 623 | 827 | 1053 | 1242 | 237 | 369 | 462 | 729 | 962 | 697 | 992 | 1289 | 1782 | 2204 |
| **Total** | 1646 | 2216 | 2657 | 3050 | 3413 | 1192 | 1807 | 2342 | 2771 | 3324 | 2838 | 4023 | 4999 | 5821 | 6737 |
